# BMI is Associated with the Willingness to Record Diet with a Mobile Food Record among Adults Participating in Dietary Interventions

**DOI:** 10.3390/nu9030244

**Published:** 2017-03-07

**Authors:** Deborah A. Kerr, Satvinder S. Dhaliwal, Christina M. Pollard, Richard Norman, Janine L. Wright, Amelia J. Harray, Charlene L. Shoneye, Vicky A. Solah, Wendy J. Hunt, Fengqing Zhu, Edward J. Delp, Carol J. Boushey

**Affiliations:** 1 School of Public Health, Curtin University, Perth 6845, Australia; S.Dhaliwal@curtin.edu.au (S.S.D.); c.pollard@curtin.edu.au (C.M.P.); richard.norman@curtin.edu.au (R.N.); J.Wright@exchange.curtin.edu.au (J.L.W.); amelia.harray@curtin.edu.au (A.J.H.); charlene.shoneye@curtin.edu.au (C.L.S.); v.solah@curtin.edu.au (V.A.S.); W.Newton@exchange.curtin.edu.au (W.J.H.); 2 Public Health Division, Department of Health in Western Australia, 189 Royal Street, East Perth 6004, Australia; 3 School of Electrical and Computer Engineering, Purdue University, West Lafayette, IN 47907, USA; zhu0@ecn.purdue.edu (F.Z.); ace@ecn.purdue.edu (E.J.D.); 4 Epidemiology Program, University of Hawaii Cancer Center, Honolulu, HI 96813, USA; CJBoushey@cc.hawaii.edu; 5 Nutrition Program, Purdue University, West Lafayette, IN 47907, USA

**Keywords:** mobile food record, novel technology, dietary assessment, young adults, overweight adults, intervention

## Abstract

Image-based dietary assessment methods have the potential to address respondent burden and improve engagement in the task of recording for dietary interventions. The aim of this study was to assess factors associated with the willingness of adults to take images of food and beverages using a mobile food record (mFR) application. A combined sample of 212 young adults and 73 overweight and obese adults completed a 4-day mobile food record on two occasions and a follow-up usability questionnaire. About 74% of participants stated they would record using the mFR for a longer period compared with a written record (29.4 ± 69.3 vs. 16.1 ± 42.6 days respectively; *p* < 0.0005). Multivariable logistic regression was used to identify those who were more likely to record mFR in the top tertile (≥14 days). After adjusting for age and gender, those with a BMI ≥ 25 were 1.68 times more likely (Odds Ratio 95% Confidence Interval: 1.02–2.77) than those with BMI < 25 to state a willingness to record with the mFR for ≥14 days. The greater willingness of overweight and obese individuals to record dietary intake using an mFR needs further examination to determine if this translates to more accurate estimates of energy intake.

## 1. Introduction

Assessing dietary intake as an outcome for interventions can be difficult as the requirement to assess changes in intake over time entails multiple data collections. The researchers need for accuracy must be carefully balanced with factors such as participant burden and reactivity bias associated with the dietary assessment methodology. A key advantage of written food records is the ability to capture whole diet and patterns of eating. Typically, written food records are undertaken for between three and seven days to capture usual intake. Increasing days of recording can lead to poorer quality records leading researchers to recommend keeping recording to seven day or less [1]. To date, food records are not recommended for evaluating dietary interventions because of the reactivity that may result from the recording process therefore magnifying the response bias [2]. The act of recording itself can lead people to alter their usual intake to simplify the recording process. This ‘self-monitoring’ effect is well known and can be beneficial for awareness raising and providing feedback where the goal is to change dietary behaviors. Many weight loss studies have used food recording as an effective behavior change strategy [3]. To date, most of the research on food records has employed written food records. Whilst it is acknowledged that further research is needed to guide best practice to assess intervention effectiveness [2], research on image-based food records for intervention research is emerging and suggests these methods may be more suitable than written food records for evaluating dietary interventions [4]. With rapid advances in technology over the past decade, written food records appear less acceptable particularly in young people who have a poor tolerance for ‘low tech’ methods [5,6]. Increasingly, nutrition researchers are needing to consider how to better engage people in dietary assessment methods. This is particularly relevant for intervention research as there is often a need to assess intake over time and on multiple occasions. Image-based dietary assessment methods have the potential to improve engagement in the task of recording making it more fun and appealing to participants [7]. An important step in this development is to obtain user feedback to inform the design of mobile methods [7,8,9].

Giving consideration to these factors, investigators have developed an image-based dietary assessment system known as Technology Assisted Dietary Assessment or TADA [10,11]. The mobile food record (mFR) App uses the camera on a mobile device to capture before and after images of food and beverages consumed. Image analysis can be either automated or undertaken by a trained analyst, thus reducing the burden on the participant [12]. Capturing dietary behavior as it occurs has the additional benefits of reducing recall bias and assessing how eating behavior varies with time and different environments [13]. There is, however, a need to evaluate how willing people are to record with image-based food records in community-dwelling settings for use in dietary interventions. The aim of this study was to assess factors associated with the willingness of adults to take images of food and beverages using a mobile food record (mFR) application running on an iPod Touch (Apple Inc., Cupertino, CA, USA).

## 2. Materials and Methods

### 2.1. Study Design and Participant Recruitment

Data were collected from two samples of adult participants and combined for this analysis. The first adult sample was drawn from a population-based sample of 247 young adults (18 to 30 years) taking part in a 6-month randomized controlled trial (RCT) to evaluate the effectiveness of tailored dietary feedback and weekly text messaging support to improve diet. The study protocol and trial outcomes have been previously published [4,14]. Briefly, participants were recruited from the Federal Electoral Roll, a compulsory enrolment system for Australians aged over 18 years. Exclusion criteria applied if people were unable to complete the 6 month study, undertaking extreme forms of exercise, on a special diet, currently studying or had studied nutrition, pregnant or breastfeeding, unable to attend the study centre to complete the face-to-face assessments or if they had any serious illnesses. A final sample size of 212 young adults who completed the 6-month study and had undertaken the 4-day mFR on two occasions were included in this analysis. The second sample was a convenience sample drawn from 118 community dwelling overweight and obese adults (20 to 67 years) participating in a 12-week RCT to determine the effectiveness of fiber supplementation on body weight and dietary intake [15]. Participants, were aged 25–70 years and BMI 25–35 kg/m^2^ were recruited through radio advertisements and through University email communication systems. Participants were excluded if they were pregnant, unable to complete the 12 week study, undertaking extreme forms of exercise or dieting, unable to attend the study centre, had an allergy to any food ingredient used in the study or if they had any serious illness or had current or recent dietary fibre supplementation. A final sample size of 73 overweight adults who completed the 12-week study and had undertaken the 4-day mFR on two occasions were included in this analysis. Both trials were registered with the Australian New Zealand Clinical Trials Registry (reference ACTRN12612000250831 and ACTRN12614000701628) and projects were approved by the Curtin University Human Research Ethics Committee (HR181/2011 and HR170/2014). All participants provided informed consent.

### 2.2. Data Collection

Both samples completed a 4-day mobile food record (mFR) on two occasions. Participants in the first sample undertook the mFR at baseline and after six months, whereas participants in the second sample, undertook the mFR at baseline and again at 12 weeks. Both samples, at their first visit had their height and weight measured and underwent identical training on how to use the mFR App for the collection of dietary information. Eating behavior was assessed at baseline using the 3-Factor Eating Questionnaire to assess cognitive restraint of eating, disinhibition and hunger [16]. Research staff conducted each training session on how to: connect to Wi-Fi for sending images; take a practice image of plastic food replicas; and send the before and after eating image pair to the back-end server. Participants were instructed to record their food and beverage intake using the mFR for four consecutive days (Wednesday to Saturday) with the investigator-supplied iPod Touch (iOS6) loaded with the mFR App. When taking an image, participants were instructed to include a provided reference object known as a fiducial marker (a checkerboard pattern of known shape, size and color) to assist with food identification and portion size estimation [11,17,18]. They were instructed to record food and beverage items not captured in an image using the iPod notes section or handwritten in a small booklet provided.

The mFR App had an automated feature to detect the presence of the fiducial marker and alerted participants if the fiducial marker was missing from the image. An angle-detection algorithm assisted participants to take the image at the correct angle for estimating the volume of food and beverages [19]. A light turned green when the angle of the mobile device was positioned between 45 and 60 degrees from the horizontal plane. The mFR performed automatic uploading of food and beverage images collected by participants when in Wi-Fi range. If participants did not have access to Wi-Fi their images were stored securely in the App until a Wi-Fi connection was made. Images extracted from the mFR were stored in the back-end server with a unique password protected participant ID entered into the mFR by the researcher [10,11,14].

A week later participants attended a second baseline visit to return the iPod Touch and complete additional written questionnaires. At this visit the researcher interviewed each participant to verify the content of the images and probe for any forgotten food and beverages. Both groups undertook a second 4-day mFR at the end of the intervention period, had their height and weight measured and completed a usability questionnaire to provide feedback on the mFR. All participants received two AUD$20 gift vouchers for participating in the studies.

### 2.3. Dietary Assessment

The researcher analyzed the 4-day mFRs using a quality scoring of food items according to the Australian Guide to Healthy Eating standard food selection guide standard servings (AGHE)) [20]. AGHE specify one standard serve of fruit as equivalent to 150 g, one serve of vegetables is equivalent to 75 g, and one serve of energy-dense nutrient-poor (EDNP) ‘junk’ foods or beverages (also referred to as discretionary food choices) as equivalent to the amount that would approximate 600 kilojoules or 143 calories). A purpose-built database was developed to capture food and beverages consumed categorized by food group, food type and serving size. Image analysis was conducted by viewing the images and manually estimating the serving size and entering the data for each day of recording. For each participant, an average serve per day was calculated for fruits, vegetables (not including fried potato), grain (cereal) foods, protein foods (lean meats and poultry, fish, eggs, tofu, nuts and seeds, and legumes/beans), milk and milk products (milk, yoghurt, cheese and/or alternatives). EDNP food and beverages were separated into junk foods, alcohol and sugar-sweetened beverages (SSB) servings.

### 2.4. Usability Questionnaire

After using the mFR on two occasions at the end of the studies, participants provided written feedback regarding their perceptions and acceptability of the mFR. To minimize data entry errors, the questionnaires were entered in duplicate by two researchers and the responses compared using the SAS compare function for mismatches. The questionnaire was composed of forced choice questions with five responses from “strongly disagree” to “strongly agree” and several open-ended questions [7,8,9]. Examples of questions relevant to this paper were: (1) Remembering to take an image before meals was easy; (2) Remembering to take an image after meals was easy; (3) Remembering to take an image before snacks was easy; (4) Remembering to take an image after snacks was easy; (5) The mFR interfered with my daily activities; (6) The mFR interfered with my social activities; (7) I found the fiducial marker (checkerboard square) easy to use; (8) I found the fiducial marker easy to carry around; (9) I found it easy to include the fiducial marker in the picture of my meals; (10) I found it easy to include the fiducial marker in the picture of my snacks; (11) Understanding the purpose of the mFR motivated me to use it; (12) Using the mFR made me behave differently while I was using it. Five open-ended questions explored the potential for dietary recording including: (1) How long would you be willing to record with the mFR App?; (2) If you were recording using paper-based food record, how long would you be willing to record? (both with response units of days, weeks and months); (3) What did you like the most about using the mFR App?; (4) What did you like least about the mFR App?; and (5) Using the mFR made me behave differently while I was using it. Additional comments and free text responses were uploaded in the NVivo 12 verbatim, coded and patterns identified using thematic analysis by two researchers (Deborah A. Kerr, Christina M. Pollard) independently. The additional comments were grouped under the five questions where participants were asked to provide open-ended responses.

### 2.5. Statistical Analysis

Data collected using the same methods among adults was used for statistical comparisons. Descriptive statistics included frequencies and percentages. The 5-category ordinal response scales used by the participants to provide feedback on the usability of the mFR App were recoded as agree, neutral and disagree. Demographic data and food group serves between the two samples were examined using the Mann-Whitney test and displayed in the tables as medians and inter-quartile-range. As the data for willingness to record were not normally distributed non-parametric tests were performed. The Wilcoxon signed rank test was used to assess significant differences between participants’ perception on their willingness to record comparing mFR with a written food record. McNemar’s test was used to compare the correlated responses to the questions ‘remembering to take an image before meals/snacks was easy’ and ‘remembering to take an image after meals/snacks was easy’. Multivariable logistic regression was used to identify the demographics of participants reporting likely to record with the mFR in the top tertile (≥14 days) compared to the rest of the sample, adjusting for age and gender. SPSS version 22 (SPSS Inc., Chicago, IL, USA) was used for all statistical analyses.

## 3. Results

Participant characteristics for the two samples are shown in Table 1. The mean age for all participants was 28.8 ± 11.2 years and ranged from 18 to 67 years. As expected, significant differences were observed between the two samples for age and BMI. For measures of eating behavior, the overweight adult sample showed significantly higher cognitive restraint (*p* < 0.001), disinhibition (*p* < 0.001) and hunger; *p* < 0.05). There was no difference in the number of days participants stated they were willing to record between the two samples. Overweight participants from both samples reported a greater number of serves for grains (*p* < 0.01) and milk and milk products (*p* < 0.01) but there was no difference for other food group servings.

### 3.1. Perceptions on the Use of the Mobile Food Record

Table 2 shows the responses in all participants regarding perceptions on the use of the mFR. Agreement with ‘remembering to take an image’ before meals was higher than before snacks (62.5% vs. 34.0, *p* < 0.0005). There were differences in agreement of remembering to take before images of meals compared with snacks (62.5% vs. 34.0, *p* < 0.0005). Agreement with ‘remembering to take an image after meals was easy’ was higher than with the statement ‘remembering to take an image after snacks was easy’ (57.5% vs. 38.2, *p* < 0.0005). Remembering to take an image before snacks appeared to be more difficult with only 34% agreeing with the statement. Less than one quarter (21.8%) of participants agreed with the statement that the mFR interfered with their daily activities and social activities (16.9%). Around 41.5% thought using the mFR made them behave differently while using it. The majority of participants (76.4%) agreed that understanding the purpose of the mFR motivated them to use it. Most regarded the fiducial marker as easy to use (93%). As the participants in the current study were provided with the mFR on an iPod they were asked about using the mFR or their own device. Seventy three percent agreed they would use the mFR more frequently if it was on their own device.

### 3.2. Willing to Record

When asked how long they would be willing to record with a food record, participants stated they would be more willing to record for more days with the mFR compared with a written food record (mean 29.4 ± 69.3 for mFR; 16.1 ± 42.6 days for written food record; *p* = 0.002). About 74% of the participants stated that they would record using mFR for longer periods compared with a written food record, and the difference between the days was statistically significant using the Wilcoxon signed rank test (*p* < 0.0005).

Table 3 shows the logistic regression analysis of demographic and usability variables associated with the likelihood of using the mFR for greater than or equal to 14 days (or top tertile), after adjusting for age, gender and BMI. One-third of the participants stated that they were willing to record for 14 days or more with the mFR. Multivariable logistic regression was used to identify the demographics of participants that were likely to record with the mFR in this top tertile or ≥14 days. After adjusting for age and gender, those with a BMI ≥ 25 were 1.68 times more likely (OR 95% CI: 1.02–2.77) than those with BMI < 25 to state a willingness to record consumption using the mFR ≥ 14 days. Factors associated with willingness to record for those with a BMI ≥ 25 compared with a BMI < 25 included ‘remembering to take an image before meals was easy’ (OR 1.97; 95% CI: 1.08–3.61) and ‘remembering to take an image before snacks was easy’ (OR 2.27; 95% CI: 1.33–3.88). Willingness to record with the mFR in those with a BMI ≥ 25 compared with a BMI < 25 was associated with being able to use the mFR on their own device (8.48 OR 95% CI: 2.48–28.96). There was no association with willingness to record by BMI for eating behavior or the amount of food group servings consumed.

### 3.3. Open-Ended Responses

#### Willing to Record with the mFR

Table 4 shows examples of responses to the open-ended questions and the main comments related to willingness to record with the mFR for both those willing to record for less than 14 days and 14 days or more. Other comments were related to difficulties with using the mFR in various situations: difficult to record ‘on the go’, at work, social situations, or when snacking. There were also comments related to recording fatigue and motivation to monitor their intake. For those who said they would be prepared to use the mobile phone record for more than 14 days, a common reason given was that it helped them monitor what they eat. The reason or motivation for recording appeared to influence the length of time they would be willing to record and if they were able to use their own device they would be more likely to do it for a greater length of time. Convenience and ease of use to fit in with their lifestyle appeared to be common themes.

For those who said they would be prepared to use the mobile phone record for 14 days or more, most of the comments regarding a written food record were that it was annoying and tedious and that the mobile phone record was easier. There was a clear preference for mobile methods among this group. There were concerns about the written food record being less accurate. Although some thought a written food record might be easier, review of themes highlighted a dietary assessment issue with written food records as their comments reflected that they would use a written food record more as a dietary recall, that is “wait till the end of the day to write down” or “can remember what I ate later” and that it was harder to remember to do it. The time and effort it would take for the written food record was also commented on.

When asked what they like most about using the mFR, the overwhelming response was that it was easy, simple and convenient. Other major themes were it made them think and it was quick. Many expressed an interest in the mFR technology. They least liked that it was hard to remember to record, limitations of the technology or glitches and using the mFR in social situations. When asked to comment if using the mFR made them behave differently the most common themes were made them think, made them more aware and influenced their snacking as they had to take frequent images ‘on the go’. This comment reflected the recording ‘at the time’ nature of the mFR versus the recall type of comments for the written food record. There were some comments regarding eating healthier and being more self-conscious, concerned or embarrassed using the mFR.

## 4. Discussion

This study found a greater willingness to record with an mFR in overweight and obese individuals. When age and gender were accounted for, those with a BMI of 25 kg/m^2^ or more were almost twice as likely to state they would be willing to record for 14 days or more (*p* = 0.042). Overweight individuals were two times more likely to agree with statements regarding ‘remembering to take an image was easy’ for before meals and before and after snacks. They were also more likely to agree that inclusion of the fiducial marker in snack images was easy (OR 2.51) and it was easy to use the mFR when away from home (OR 2.05). Those with a higher BMI showed a much greater willingness (OR 8.48) to record with the mFR if it could be installed on their own device, suggesting convenience and ease of access were important considerations for future use. Of significance, although the overweight adult sample scored higher for measures of eating behavior such as cognitive restraint, disinhibition and hunger, these factors were not associated with willingness to record. A high cognitive restraint can indicate the conscious effort to restrict foods perceived as high in calories. In comparison to the young adults, the overweight adults showed no difference in the reporting of food group serves (e.g., junk foods, sugar-sweetened beverages and alcohol) typically underreported with dieters or restrained eaters.

The finding that those with a higher BMI were more willing to record is unexpected and highlights the gap in knowledge in understanding the psychological factors that influence the recording process. Hebert [21] in a recent editorial identified the need for inclusion of response sets such as social desirability, social approval and other psychological traits as part of routine dietary data collections. Our findings would support this recommendation but also suggest for the mFR there may be a variety of factors influencing people’s willingness to record. Although we obtained a measure of eating behavior, social desirability and social approval were not assessed. Social desirability is described as the tendency to respond in a manner to avoid criticism whereas with social approval people respond in a manner to seek praise [22,23]. These response biases are known to differ by gender, education and dietary assessment method [24,25] but to date these factors have not been assessed in association with image-based methods.

About 41% of individuals thought they behaved differently when using the mFR App, suggesting some reactivity may have occurred. Reactivity, described as a change in behavior due to the awareness of the behavior being measured [2] can be perceived as either positive or negative. Participants commented that undertaking the mFR made them more aware or made them think about what they were eating. This change in behavior due to the act of recording is a common finding with both paper-based [1,26] and technology-based methods [27]. With technology-based methods little has been published however, in a study of 25 pregnant women where diet was assessed using an Evernote^®^ App (Mobile and desktop app software, 2016 Evernote Corporation, Redwood City, CA, USA), women commented that the act of recording had a positive influence on their eating but some women felt guilty when taking images of foods such as sweet biscuits and chocolates [27]. Participants in the current study mostly viewed being made more aware as positive but for some reported feeling embarrassed or more self-conscious of their food intake. This negative effect was highlighted in comments on what they like least about using the mFR App. Some participants commented they had difficulty using the mFR in social situations and didn’t like drawing attention to themselves. This effect seemed to be relatively minor overall as less than one quarter of participants thought the mFR interfered with their daily activities and social activities.

Self-monitoring is recognized as a successful weight loss strategy and is likely to work by raising awareness of dietary behaviours [3]. The greater willingness to record in those with a higher BMI with the mFR may indicate these adults were able to recognize the potential benefits of self-monitoring in helping them lose weight. Carter et al. [28] using a digital data entry App designed for a weight loss intervention, found those with the highest frequency of monitoring lost the most weight. However, in a previous 6-month dietary intervention in young adults, control group participants did not lose weight as a result of undertaking the mFR on two occasions. These findings suggests self-monitoring with the mFR alone will not induce weight loss [4]. Further exploration would be needed to evaluate if the mFR App could be adapted as a tool for self-monitoring purposes. Participant burden is a concern common to all dietary assessment methods as it impacts on the acceptability and feasibility of these methods, and hence on the completion rates and reliability of the resultant data. When asked what they liked most about using the mFR the most frequent response was it was ‘easy to use’ and ‘quick’, suggesting these are essential considerations for acceptability of mobile methods. Less than a quarter of participants thought using the mFR interfered with their daily activities or social interactions. Participants stated that what they liked least about the mFR App was having to remember to use it or that it was inconvenient when busy. These findings suggest that researchers need to consider the lifestyles of participants and the situations they may find challenging to take images such as at work, ‘on the run’ or when in public. Previous training of adolescents in how to use the mFR included interactive sessions designed to address potentially problematic scenarios such as being on the bus or in a movie theatre [8]. The study found that after training the adolescent’s perceptions around ease of use increased significantly. Therefore incorporating some of the problematic scenarios identified in this study into future user training may improve the acceptability of the mFR in these situations.

Perceptions regarding the ease of use and convenience were also important in participant’s willingness to record with the mFR. This finding was consistent across all participants. Participants also commented they tended to snack less because of the inconvenience of taking images for small items. This is an interesting finding as this suggests that a meal is worth taking an image but a small snack isn’t; whether this differential completion applies differently across different modes of data collection is an area of potential future research. People may have also been less inclined to take images of their snacks due to social desirability bias or for fear of a negative evaluation [29]. This is consistent with an early qualitative study of participants’ experience with written food records where they expressed concerns about being judged [26]. Seventy three percent of participants agreed they would use the mFR more frequently if it could be installed on their own device. In the current study, participants carried their own device and a study iPod Touch with the mFR App installed. From these results the capability for installation on participants own device that is now available for android and iPhone devices may further enhance the acceptability of the mFR App. Understanding the purpose of the mFR also appeared to be a strong motivation with 76% agreeing that this would motivate them to use it. The training of participants included background on the mFR App, its purpose and how it worked. This appears to have assisted with engaging the participants in the task of recording. Some respondents expressed an interest in the mFR technology, but they disliked glitches and where there were extra steps they didn’t think were needed. For example, as the mFR requires a before and after eating image, participants who ate everything became annoyed at having to take the after image every time.

The use of the fiducial marker didn’t appear to be viewed at all negatively by participants. There was very high agreement regarding the use of the fiducial marker with 93% agreeing with the statement ‘I found the fiducial marker easy to use’ and easy to carry around (82% agreement). When taking an image, participants were instructed to include the fiducial marker to assist with food identification and portion size estimation [12,13,14]. As the training included an explanation on the purpose of the fiducial marker this may have assisted with the acceptability of this in the study.

In this study there was a greater acceptance of recording with the mobile food record compared with a written food record with about 74% of participants stating they would be willing to record for longer using the mFR compared with a written food record (*p* < 0.0005). Written records were viewed as annoying, tedious, time consuming and not as accurate. This is consistent with the findings of Hutchesson et al. who found in young women, a written food record was the least preferred method compared with computer or smartphone due to inconvenience and time [30]. Similarly adults with type 2 diabetes expressed a preference for an image-based method over a written food record [31]. Some individuals in the current study indicated a preference for a written food record. They considered this method would be more convenient as they could complete the written record as a recall at a more convenient time. This is consistent with our previous work in adolescents where they indicated they would recall their intake rather than record it at the time [5]. Collectively these findings stress the importance of developing methods that fit in with people’s lifestyles. Researchers need to balance the pursuit of accuracy with the individual’s desire for a method that is quick and easy and minimization of the interruption to their daily lives. Some commented on recording fatigue and had problems being able to maintain interest once it was no longer a novelty. The experience of using the mFR App needs to be fun and engage participants in the activity.

There were several limitations with the current study. Measures of response bias, such as social desirability scales were not undertaken but it is recommended that future studies with the mFR should focus on identifying the psychological constructs related to willingness to record with the mFR. One limitation is that the sample have all experienced the mFR on two occasions but had not all undertaken written food records and this may have biased their perceptions of what completing a written food record entails. Although participants recruited were from a diverse background for socio-economic status and ethnicity, the response rate in both samples was higher from women than men, consistent with other population studies in Western Australia [32]. Potentially, there may have been other factors, such as differences in recruitment and study requirements between the two study samples that were not accounted for in examining participants’ willingness to record with the mFR. Although this study showed a greater willingness to record with the mobile food record by people with a higher BMI, it remains to be tested if this will lead to more accurate reporting. Future studies in large ethnically diverse populations should obtain independent measures of underreporting such as doubly labelled water.

## 5. Conclusions

This study aimed to assess factors associated with the willingness of adults (18–68 years) to take images of food and beverages using a mobile food record (mFR) application. Participants who had recorded their food and beverage intake for a 4-day period using the mFR on two occasions showed a high acceptance of the method. There was a strong preference for a mobile food record over written food records. Being able to use the mFR on their own device and remembering to take image of meals and snacks was associated with greater willingness to record for those with a BMI ≥ 25 compared with a BMI < 25. The greater willingness to record dietary intake with an image-based mobile food record in those with a higher BMI needs further exploration, particularly a better understanding of the motivations to record and if these findings will translate to more accurate estimates of energy intake.

## Figures and Tables

**Table 1:** Characteristics of participants comparing the young adult sample with the overweight adult sample testing the usability of the mobile food record.

Variables at Follow up	Young Adults (*n* = 212)	Overweight Adults (*n* = 73)
Women	144 (67.9%)	51 (69.9%)
Men	68 (32.1%)	22 (30.1%)
Age (years), median (IQR) ^4^	24.0 (21.0–27.0)	39.7 (27.9–57.5) ^3^
Weight (kilograms), median (IQR)	66.1 (58.5–79.2)	76.9 (69.0–89.8) ^3^
Height (metres), median (IQR)	1.68 (1.62–1.76)	1.65 (1.61–1.73)
BMI (kg/m^2^), median (IQR)	23.3 (20.9–26.0)	27.8 (25.7–30.8) ^3^
	BMI Categories	
BMI < 25	143 (67.5%)	14 (19.2%)
BMI overweight (≥25, <30)	50 (23.6%)	35 (47.9%)
BMI obese (>30)	19 (9%)	24 (32.9%)
	Ethnicity	
White	162 (76.4%)	49 (67.1%)
Asian	37 (17.5%)	23 (31.5%)
Other	13 (6.1%)	1 (1.4%)
	Eating Behavior median (IQR)	
Cognitive restraint	7.0 (5.0–12.0)	12.0 (9.5–15.0) ^3^
Disinhibition	6.0 (4.0–8.5)	9.0 (7.0–11.0) ^3^
Hunger	6.0 (4.0–8.0)	7.0 (5.0–9.0) ^1^
	Number of Days Willing to Record median (IQR)	
Mobile food record	7 (7–28)	7 (4–21)
Written food record	7 (3–14)	5 (2–7)
	Food group serves average/day median (IQR)	
Vegetables	2.0 (1.3–2.9)	2.4 (1.8–3.5) ^1^
Fruit	0.6 (0.3–1.3)	0.6 (0.1–1.3)
Junk foods	2.4 (1.4–3.6)	2.5 (1.4–3.9)
Sugar-sweetened beverages	0.1 (0.0–0.6)	0.0 (0.0–0.1) ^3^
Alcohol	0.0 (0.0–0.7)	0.0 (0.0–0.5)
Total EDNP serves ^5^	3.3 (1.9–4.5)	2.9 (1.9–4.6)
Grains	2.8 (2.0–3.8)	3.5 (2.4–4.8) ^2^
Protein group	1.6 (0.9–2.3)	1.6 (1.1–2.1)
Milk and milk products	0.9 (0.5–1.4)	1.3 (0.8–1.9) ^2^

^1^ *p* < 0.05, ^2^ *p* < 0.01, ^3^ *p* < 0.001 significantly different by Mann-Whitney test; ^4^ inter-quartile range; ^5^ Total energy-dense nutrient poor food group serves includes junk foods, sugar-sweetened beverages and alcohol.

**Table 2:** Comparison of perceptions for adults (*n* = 285) regarding the use of the mobile food record (mFR) app for 4 days.

Statements Regarding the Use of the mFR App	Responses, *n* (%)		
	Strongly Agree or Agree	Neither Agree or Disagree	Disagree or Strongly Disagree
Remember to take an image was easy:			
Before meals	178 (62.5%)	39 (13.7%)	68 (23.9%)
After meals	164 (57.5%)	43 (15.1%)	78 (27.4%)
Before snacks	97 (34.0%)	44 (15.4%)	144 (50.5%)
After snacks	109 (38.2%)	55 (19.3%)	121 (42.5%)
Using the mFR:			
Interfered with my daily activities	62 (21.8%)	80 (28.1%)	143 (50.2%)
Interfered with my social interactions	48 (16.9%)	77 (27.1%)	159 (56.0%)
Made me behave differently while I was using it	118 (41.5%)	58 (20.4%)	108 (38.0%)
Understanding the purpose of the mFR motivated me to use it	217 (76.4%)	53 (18.7%)	14 (4.9%)
It was easy to use the mFR when away from home	199 (69.8%)	36 (12.6%)	50 (17.5%)
If the mFR was on my own phone I would use it more frequently	208 (73.2%)	47 (16.5%)	29 (10.2%)
I found the fiducial maker:			
Easy to carry around	234 (82.1%)	21 (7.4%)	30 (10.5%)
Easy to include in the picture of my meals	248 (87.0%)	20 (7.0%)	17 (6.0%)
Easy to include in the picture of my snacks	228 (80.0%)	25 (8.8%)	32 (11.2%)
If it could fit easily in my pocket I would find it easy to carry around	213 (75.0%)	40 (14.1%)	31 (10.9%)

**Table 3:** Odds ratios of the likelihood of using the Mobile Food Record (mFR) for 14 days or more (*n* = 119) compared to less than 14 days (*n* = 161) among the young adult and overweight samples ^1^.

Variable	Odds Ratio (95% CI)	*p*-Value
After adjusting for age and gender:		
BMI (≥25 kg/m^2^ versus <25 kg/m^2^)	1.68 (1.02–2.77)	0.042
After adjusting for age, gender and BMI ^1^:		
Remembering to take an image before meals was easy	1.97 (1.08–3.61)	0.027
Remembering to take an image before snacks was easy	2.27 (1.33–3.88)	0.003
Remembering to take an image after snacks was easy	2.63 (1.51–4.58)	0.001
I found it easy to include fiducial marker in the pictures of my snacks	2.51 (1.08–5.84)	0.033
It was easy to use the mFR when I was away from home	2.05 (1.05–4.00)	0.035
If I could use the mFR on my own mobile I would use more frequently	8.48 (2.48–28.96)	0.001

^1^ willingness to record for 14 days or more represents top tertile compared to the remainder of the sample; ^2^ comparing strongly agree or agree to, neither agree or disagree and strongly disagree or disagree as recorded on the usability questionnaire.

**Table 4:** Open-ended responses of adults regarding the use of the mobile food record comparing those who would be willing to record with the mFR for less than 14 days with those willing to record for 14 days or more.

Themes	Examples of Comments Would Record for <14 Days	Examples of Comments Would Record ≥14 Days
**How long in days/weeks/month would you be willing to record what you eat using the mFR?**		
Remembering to use	“*Only because I’m hopeless at remembering to record the food as my routine is always different. And I have the worst memory ever.*” “*It was difficult to remember taking photos before and after meals especially when out and about or on the go. I missed many meals and although I'd try for as long as need be, ultimately I would forget to record many things.*”	“*I think a month would provide a more consistent, overall picture of my usual eating habits. I also might do better with remembering to take photos. :)*” “*I found that the business of the day greatly affected my ability to remember and willingness to use the CHAT app. When you are eating on the go it is difficult to remember/take the time.*”
Motivation to monitor	“*Really like the "taking a photo" idea. Maybe improve graphics of camera.*” “*It was easy to use but when going out is a bit inconvenient. The good point of using this is might reduce the unnecessary food or snack due to lazy to snap the photo.*”	“*Think a month would provide a more consistent, overall picture of my usual eating habits. I also might do better with remembering to take photos. :)*” “*A little more inconvenient, so I’d only really do it for a good reason.*”
Difficult to record	“*I snack quite a bit on the move so it's difficult to keep taking pictures.*” “*Gets a little bit hectic and inconvenient at times, especially given how rarely I eat at home.*”	“*Depends on work condition or shifts. Found hard to remember on night shifts because eating pattern is out.*” “*Difficult to record food when in work lunch situations-didn’t want to draw conversation away from what we were discussing. Always had trouble remembering to take shot after meals.*”
Recording fatigue	“*4 days is more than enough or else it would be too boring to do it all the time.*”	“*That’s probably about as long as it could keep my interest. Beyond that I'd start to forget just because it's no longer a novelty.*”
**How long would you be willing to record what you eat using a totally paper-based food record?**		
Accuracy	“*I hate paper based stuff, this was still interesting. Also paper based food record will not be as accurate as this in my view point.*”	*“Might make it easier to get a record in social situations, but would be hard to estimate quantity correctly.” “Easier to remember what you’ve eaten and record it down later—but then might not be as accurate!”*
Annoying and tedious	“*I just wouldn’t be bothered*”. “*Pen and paper is not great for me, always losing paperwork also my kids rip them up.*” “*Would rather not…*” “*I have no patience for paper recording. Phone all the way.*”	*“I would get annoyed with writing all down and lose interest, taking photos was much easier. =)”.*
Prefer to recall not record	“*It is easier as I don't have to worry about taking a photo rather I could write it down when I remember.*” “*I find this much easier and socially acceptable for me. I can recall what I have eaten and write it down at a suitable time.*”	“*I think even recording what you eat using notes on the iPod is better because you can fill out your whole day’s intakes in one go.*” “*Easier in a way because you can add things you forget later—this is what I do with the Fitness pal app sometimes.*”
Time	“*I eat a lot and don’t have the time to write it all down especially at work*.”	“*Oh Woah, paper is much more paper work! It's not suitable for busy people, if the form is complex.*” “*Painful!! Time consuming.*”
List what you liked most (if anything) about using the mFR		
Easy, simple, convenient	“*Easy to use. Helpful in dietary awareness. A good, highly creative idea.*” “*Very easy, even enjoyable. It makes you pay attention what and how much you eat.*”	“*Incredibly easy to use and so easy to take pictures of my food using the guide on the screen.*” “*Easy to use. Like taking photos.*”
Made me think	“*Made me a little more mindful about what I was eating/drinking.*” “*I would put off having a snack because I did not want to go through the hassle of the before and after photos. I liked that it made me think about what I was eating.*”	“*I think taking the picture makes you realize that it’s not really healthy.*” “*It was easy to use and made me ‘realize’ what kind of foods I was eating.*”
Quick	“*Fast!*” “*Not needing to write all the things eating so is quicker then writing.*”	“*Quicker and easier to take a photo, rather than writing down food intake and guessing quantity and weight*”. “*Quick and easy to use and send data.*”
Interest in the mFR technology	“*Easy to use. Helpful in dietary awareness. A good, highly creative idea.*” “*The kids thought it was fun.*”	“*I really liked that it could tell the angle of each shot when taking a picture. All I had to do was press the button.*” “*It’s quite quirky and a funny thing to do—for a while.*”
List what you liked least (if anything) about using the mFR		
Remembering to use	“*To remember using it every single time, snack especially.*” “*Found it annoying to use and easy to forget about. Found snacking more difficult as food had to be individual rather than say snacking from a combined bowl, jar, etc.*”	“*Inconvenient when I was busy and rushing to eat my meal (I would then forget to take a picture of my meal).*” “*I wish it had had a daily reminder that was loud, or sent to my phone! And possibly more than once a day.*”
mFR capabilities and technology glitches	“*There were a few glitches with the app, but apart from that it was good.*” “*Having to take ‘after’ shots when I’d eaten everything. No ability to add notes to individual images. Prompting to send every image individually.*”	“*It was sometimes difficult to get the correct lighting.*” “*Just the glitch with taking the pics became a little annoying!*”
Social situations	“*Using it in social situations/work to take photos of the food. Made the interaction unnatural and this made me uncomfortable. I would forget or avoid taking the photo.*” “*Felt a bit silly taking photos of my food in public—i.e., café’s, work tea room.*”	“*Kinda awkward to take picture when you are with friends especially a squeezy table with foods packed.*” “*Sometimes it draws attention at dinner/social gatherings, but it's not too bad.*”
Using the mFR made me behave differently while I was using it		
Made me think	“*Made me think twice about what to eat especially on the road.*” “*I always thought about what I was having generally snack food. I also didn’t dine at certain places because of the difficulty of taking pictures i.e., Sushi train, Asian, etc.”*	“*Using the app makes you think about choosing better foods (but as you can see long weekend style, I did eat what I norm eat except McD, I hate it but everything else was closed).*”
Made me more aware	“*I was more aware at everything I was eating, so whilst I didn’t make efforts to ‘perform’ for the app, my subconscious was likely being more active in the selection process, (or it was just to annoying to take a photo in the rush and skipped a snack.*”	“*I really cut down on my snacks, (1) because it was an effort to take the picture; (2) because it made me realize I was eating due to boredom.*”
Less snacking	“*Less snacking (merely cannot be bothered). Somewhat self-conscious in public regarding taking photos of my food.*”	“*Using the app was an extra step so it discouraged me from eating smaller snacks (I had to think about whether I wanted the snack enough to justify using the app).*”
Eating healthier	“*Felt compelled to eat healthier.*”	“*In a way I felt ohh I should eat something healthy. :).*”
Self-conscious and self-concern	“*Made me feel exposed in front of friends/colleagues. Was a talking point every time I took a photo.*”	“*When you know someone will see what you eat it is easy to become embarrassed and try to eat better. I think this would disappear the longer someone uses the app.*”
